# Normalizing Electrocardiograms of Both Healthy Persons and Cardiovascular Disease Patients for Biometric Authentication

**DOI:** 10.1371/journal.pone.0071523

**Published:** 2013-08-20

**Authors:** Meixue Yang, Bin Liu, Miaomiao Zhao, Fan Li, Guoqing Wang, Fengfeng Zhou

**Affiliations:** 1 Shenzhen Institutes of Advanced Technology, and Key Lab for Health Informatics, Chinese Academy of Sciences, Shenzhen, Guangdong, P.R. China; 2 Key Laboratory of Zoonosis, Ministry of Education, Norman Bethune College of Medicine, Jilin University, Changchun, Jilin, P.R. China; 3 First Hospital of Jilin University, Changchun, Jilin, P.R. China; University of Catania, Italy

## Abstract

Although electrocardiogram (ECG) fluctuates over time and physical activity, some of its intrinsic measurements serve well as biometric features. Considering its constant availability and difficulty in being faked, the ECG signal is becoming a promising factor for biometric authentication. The majority of the currently available algorithms only work well on healthy participants. A novel normalization and interpolation algorithm is proposed to convert an ECG signal into multiple template cycles, which are comparable between any two ECGs, no matter the sampling rates or health status. The overall accuracies reach 100% and 90.11% for healthy participants and cardiovascular disease (CVD) patients, respectively.

## Introduction

Biometric features have been widely used in multiple identity authentication areas, *e.g.* access control system and communication authentication, etc [1]–[5]. The biometric features used for authentication include fingerprint, face and voice [6]. It was demonstrated that the integration of multiple biometric features could significantly increase the authentication accuracy [7], [8]. However, these individualized features have the possibility to be duplicated or faked to bypass the authentication system. So other more secure individualized features are sought for the biometric authentication problem. The electrocardiogram (ECG) signal is individual-specific in the sense of amplitude, peak and other characteristics, and difficult to be faked [9], [10]. ECG also represents a versatile factor that can derive a number of personal health measurements, *e.g.* dynamic heart rate variation, and acts as the diagnostic basis of various cardiovascular diseases (CVDs), *e.g.* arrhythmia [11], [12]. So it is becoming one of the major new features for the biometric authentication problem [1], [6], [9], [10], [13].

The major problem of ECG-based biometric applications is the dynamic temporal changes of ECG signals due to the physiological activities of the human subject. The majority of currently available algorithms extract measurements between the peaks and valleys of ECG signals. Generally, ECG based biometric algorithms can be categorized as fiducial point dependent or independent, according to whether they detect the ECG sensor's physical position on the subject body [14], [15]. The ECG-based biometric authentication (EBA) problem for healthy persons can be satisfyingly solved with accuracy> = 95%, but a much lower accuracy may be achieved for CVD patients. A discrete wavelet transformation algorithm achieved accuracies of 100% and 81% on 35 healthy persons and 10 arrhythmia samples, respectively [16]. The discrete cosine transformation based algorithm achieved accuracies of 84.61% and 100% on 13 healthy samples on the PTB and MIT-BIH databases, respectively [10]. Another recent study applied the cross correlation algorithm on the EBA problem, and achieved 80%, 70% and 80% in accuracies on the chosen 10 arrhythmia samples from the databases AFPDB, SVDB and TWADB, respectively [17]. Furthermore, most of the current algorithms only work well when the training and testing data were detected by the same experiment [18].

With the increased algorithm accuracy of the EBA problem, this technique is becoming more of interest to industries in biomedical engineering and mobile personal identification. Min *et al* filed patents KR2006082677-A and KR750662-B1 in 2007 to recognize different persons based on the limb lead III ECG signal [19]. Sun *et al* proposed a biometrics information system to store and measure biomedical signals including electrocardiograms in patent KR2011099197-A in 2011 [20]. And Apple Inc recently also filed a patent (20100113950) for an ECG-based biometrics application [21].

We propose in this study a novel normalization and interpolation algorithm to transform the ECG signal of any person collected at any time into a comparable template ECG cycle (TEC). The algorithm features a high similarity among the TECs of the same person, either healthy or with cardiovascular disease, but a much lower similarity between two TECs of different persons. This study also represents one of the most comprehensive investigations of the EBA (ECG-based biometric authentication) problem. A real-world EBA problem with no prior knowledge of sample health status was also investigated in a mixture of healthy persons and CVD patients. A consistently high accuracy suggests that our algorithm could greatly facilitate the EBA model in clinical cases. Finally we discussed two potential future directions for improving our algorithm.

The major contributions of this study include a description of the first biometric algorithm based on direct comparison of the ECG curve itself, and the consistently high accuracies of this algorithm on different data sources, which is a major problem faced by the other algorithms.

## Materials and Methods

### Data sources and preprocessing

The ECG data of 52 healthy persons were downloaded from the PTB database in the PhysioNet Diagnostic ECG Database [22]. Abnormal ECG data of 105 persons (including 14 healthy persons and 91 patients with cardiovascular diseases (CVDs)) were downloaded from the QT database in PhysioNet [23]. Database QT mainly consists of ECG signals with varied QT or ST intervals, including arrhythmia [23], [24]. Both datasets were downloaded on March 5, 2012. The list of sample IDs used in this study can be found in Table S1. The ECG signals were de-noised using a 0.5–45 Hz Butterworth Band-Pass (BBP) filter [25].

Given an ECG signal *E*, we detect the R waves using the So-Chan method [13], and split *E* into ECG cycles between two neighboring R waves. Although some studies generated the ECG cycles with fixed-length flanking regions of R waves, it's possible to include multiple neighboring R waves in one cycle, due to the varying heart rates. Considering the high accuracy in detecting R waves, this study considers the region between two R peaks as one ECG cycle.

### ECG cycle interpolation (ECI) algorithm

Two normalized ECG cycles are not comparable due to the following two reasons. Firstly, the two cycles may be detected using different sampling frequencies, which make them have different numbers of data points. Secondly, even with the same sampling frequency or within one ECG signal, the two cycles may have different lengths due to various reasons, e.g. running or arrhythmia. This also makes the two cycles have different numbers of data points.

We interpolate the normalized ECG cycle by fitting it with a cubic spline curve between any pair of neighboring data points , as similar in [9]. Given the normalized ECG cycle with 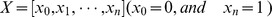 and 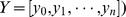, the cubic spline curve 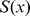 is defined as:
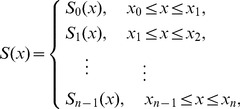
(1)where 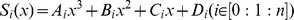, 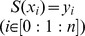, 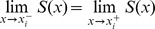, 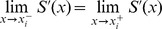, and 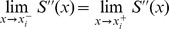 where 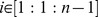.

The interpolation interval is 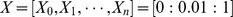, and the interpolated ECG cycle is 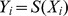, where 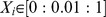. In this study, the difference between two ECG cycles is measured by the Euclidean Distance (*EucDist*) [26] of their normalized and interpolated vectors. The Euclidean Distance between two vectors 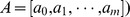 and 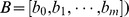 is defined to be:
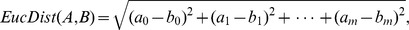
where the smaller the *EucDist* between two ECG cycles is, the more similar the two ECG cycles are to each other.

### ECG cycle normalization (ECN) algorithm

Given an ECG cycle 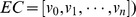 with the sampling times 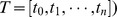, let the minimum and maximum voltages (in *mV*) of 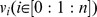 be 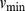 and 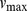, respectively. *EC* is linearly scaled by the following formula:
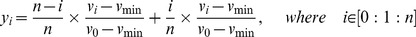
(2)


The sampling times are also linearly scaled to be between 0 and 1, as follows:
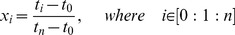
(3)


### ECG cycle normalization and interpolation algorithm (ECOIL)

**E**CG **C**ycle N**o**rmalization and **I**nterpolation A**l**gorithm (ECOIL)

**Input:** an ECG signal *E*

**Output:** an ECG cycle *M* with its data points sampled at 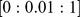 and the curve within 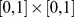.

### Procedure:

1. Denoise *E* with the BBP filter;
2. Detect the *R* waves in *E*;
3. Split *E* into ECG cycles between neighboring R waves;
4. Interpolate the ECG cycles using the algorithm ECI;
5. Normalize the ECG cycles using the algorithm ECN;
6. Cluster the cycles into two clusters based on the pair-wise Euclidean distances, using the *k*-means clustering algorithm;
7. Randomly choose 10 cycles 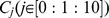 from the larger cluster;
8. For each 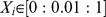, calculate the medium value 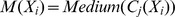, where 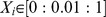 and 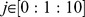.
9. Output the ECG cycle 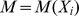, where 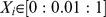.

**Figure 1:**
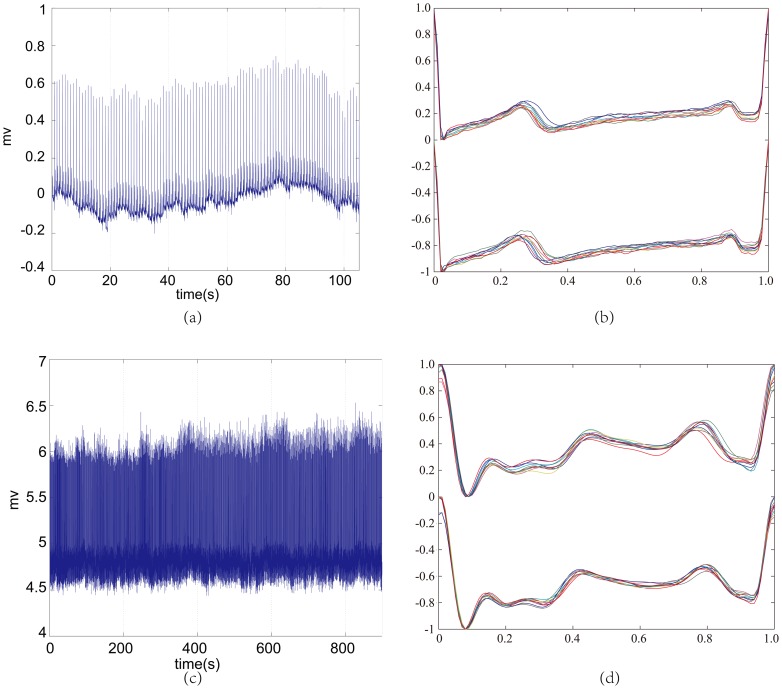
The ECG curves of the samples s0306lre and sel100 m, respectively. (a) The original curve and (b) the 10 representing training and testing ECG cycles of s0306lre from the PTB database. And the same datasets (c) and (d) for sel100 m from the QT database. For (a) and (c), the horizontal axis is in seconds, and the vertical axis is in mV.

**Figure 2:**
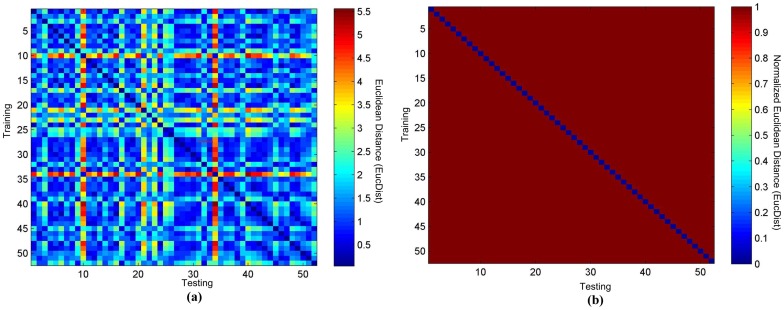
The heatmaps of TEC matching of 52 healthy persons. (a) The original heatmap and (b) the binary heatmap.

**Figure 3:**
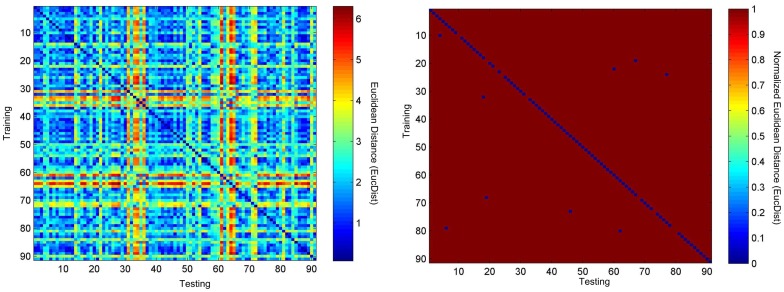
The heatmaps of TEC matching of 91 CVD patients. (a) The original heatmap and (b) the binary heatmap.

**Figure 4:**
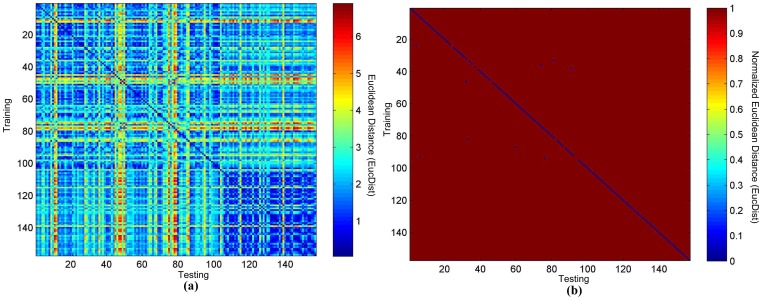
The heatmaps of TEC matching of 52+14 = 66 healthy persons and 91 CVD patients. (a) The original heatmap and (b) the binary heatmap.

We derive a template **E**CG **c**ycle (*TEC*) from an ECG signal by the above procedure. The random choice of 10 representative TECs is based on the observation that the majority of the generated TECs are highly individual-specific, as shown in Figure 1 (b) and (d). The classification performance of the 10-TEC based biometric algorithm also suggests that the choice of a limited number of TECs is both consistently accurate and calculation efficient, as shown in Figures 2, 3 and 4.

### Performance evaluation strategies

We investigated the ECOIL algorithm on solving the biometric authentication problem, which seeks a differentiating measurement of two biometric data, so that the data from the same person are more similar to each other than to those from different ones. We measured the difference between two ECG signals by the Euclidean Distance (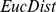) of their TECs. The function 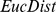 is defined in the above section of *ECG Cycle Interpolation (ECI) algorithm*. The larger the 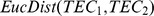 is, the less similar the two ECG signals are to each other.

An ECG signal was split into two halves with equal lengths, and the training and testing TEC data of this ECG signal were generated on the first minute of each of the two halves. There were denoted as 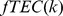 and 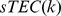, respectively , where 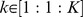. Some ECG data in the PTB database are shorter than two minutes, and the training and testing TECs were generated from the full first and second halves of the data. Next we built the template library 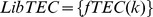, where 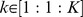. The identity of a testing data 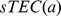 is defined as:
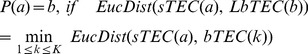
(4)


The numbers of both training and testing datasets are 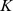, and the number of persons with correct identity predictions is 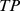. The testing dataset can be regarded as an independent test set, because the training and testing data of the same persons were collected at different times. The algorithm performance was evaluated by the accuracy 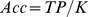. The ECG-based biometric authentication (EBA) problem consists of the training dataset 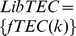, and testing dataset 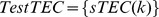. A prediction of 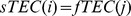 is correct, only if both TECs come from the same person, *i.e.* the label of host 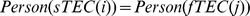. The label of each person represents a class, and there are 52 and 105 classes for the EBA problem on the PTB and QT databases, respectively. Since classification measurements, sensitivity and specificity, can only be calculated on a binary classification problem, only recognition accuracy is investigated in this study.

Due to the fact that the ECG-based biometric authentication problem determines which sample in the training dataset a query signal belongs to, we did not train on one dataset, and test on another, with no overlapping samples between the two datasets.

## Results and Discussion

### Self similarity of the processed ECG cycles

We investigated the self similarity of the ECG cycles before and after the processing of the ECN and ECI algorithms, using the samples s0306lre and sel100 m from the PTB and QT databases, respectively. For sample s0306lre, the voltage of the ECG cycle baselines varies with sampling time, as shown in Figure 1 (a). The 10 representative training ECG cycles after the ECN and ECI algorithms perfectly fit each other, as in the top part of Figure 1 (b). The 10 representative testing ECG cycles fit with each other, too, as in the bottom part of Figure 1 (b). And there is also a very high similarity between the 10 training and 10 testing ECG cycles. As for QT sample sel100 m, the voltage of the QRS waves varies with sampling time, but similar consistency can still be observed in the ECG cycles processed by the ECN and ECI algorithms, as shown in Figure 1 (c) and (d). The same pattern holds for all the other samples in the PTB and QT databases.

### ECG-based biometric authentication of healthy persons

Firstly, we studied the similarities of the TECs from the testing dataset to the training dataset of 52 healthy persons in the PTB database. As shown in Figure 2, the colors blue and red represent the smallest and largest *EucDist* between two TECs. The heatmap shows a consistently matching TEC from the testing dataset to that of the same person in the training dataset. To make the illustration clearer, we changed all the values but the smallest one in each column to 1, and the smallest value in each column to 0, which makes the heatmap have two colors, called a binary heatmap, as shown in Figure 2 (b). Figure 2 (b) clearly shows the accuracy is 100% for the PTB database using the ECG signals of 52 healthy persons.

### ECG-based biometric authentication of CVD patients

**Figure 5:**
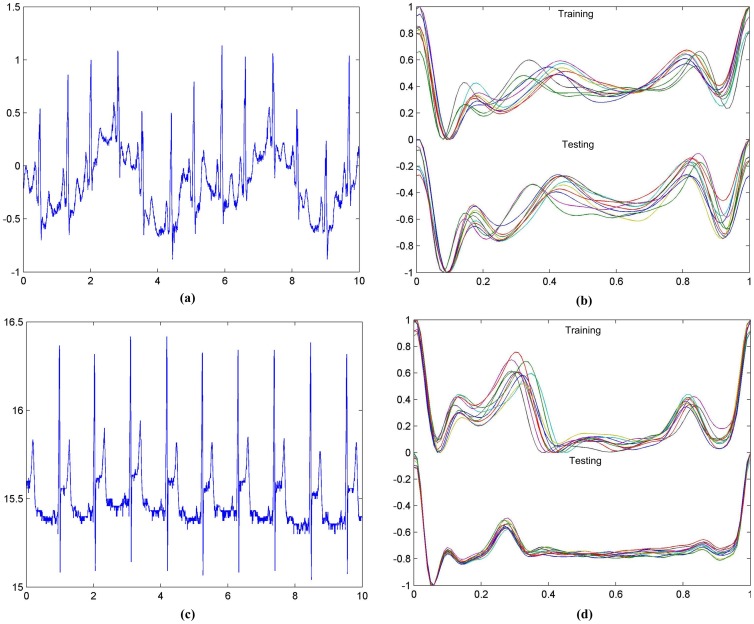
The ECG signal and processed ECG cycles of two samples. (a)–(b) sel39 m, and (c)–(d) sele0136 m. The ECG cycles in the first 10 seconds of the two signals were plotted, to make a clearer curve of the two signals. For (a) and (c), the horizontal axis is in seconds, and the vertical axis is in mV.

We further evaluated the similarities of the TECs from the testing dataset to the training dataset of 91 CVD patients in the QT database. As shown in Figure 3 (a), there is a consistent self similarity between the training and testing TECs of the same patient. The accuracy 90.11% (∼82/91) suggests that our algorithm outperforms the others on the CVD patients by at least 10%, as shown in the binary heatmap in Figure 3 (b) [16], [17]. The incorrect predictions for some samples could be due to the significant shifting of P and T waves between different cycles of ECGs, as shown in Figure 5 (a)–(d).

### Combined performance of both healthy persons and CVD patients

We also produced one dataset from the ECGs of all the 52 healthy persons in the PTB database, and 14 healthy persons and 91 CVD patients in the QT database. Since the two databases, *i.e*. PTB [22] and QT [23], were independently collected and curated, we believe that this dataset of mixed samples well represents a practical ECG-based biometric authentication problem. Next, we investigated the biometric authentication accuracy of the 157 persons, with no prior knowledge about the diseases of the persons for the algorithm. Only the same 9 CVD patients received incorrect predictions, and our algorithm produced a satisfying accuracy of 94.27% (∼(157–9)/157). Hence our algorithm works reasonably well on independently collected datasets, and does not produce confused results among different datasets.

### Longer signal duration increases authentication accuracy

**Figure 6:**
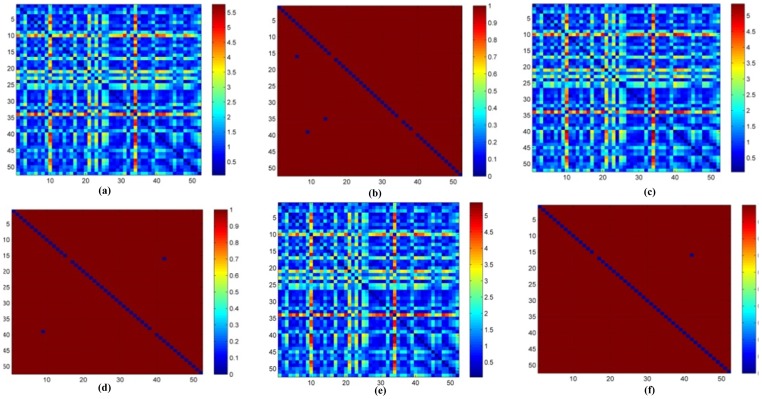
The heatmaps of TEC matching of 52 healthy persons in PTB database. (a) The original heatmap with 5 s and (b) binary heatmap with 5 s. (c) The original heatmap with 10 s and (d) binary heatmap with 10 s. (e) The original heatmap with 20 s and (f) binary heatmap with 20 s.

We further investigated whether a shorter ECG signal duration produces a reasonable biometric authentication accuracy, based on the PTB database. As illustrated in Figure 6, when the ECG signal duration is 5, 10 and 20 seconds, the numbers of incorrect authentication cases are 3, 2 and 1, respectively. Overall authentication accuracies are 94.23%, 96.15% and 98.07%, respectively. The data in Figures 2 and 6 suggests that a longer ECG signal detection time leads to better ECG-based biometric authentication accuracy. Considering that the rest ECG signal is usually taken for a period of 10 seconds, *e.g.* in an office visit during an annual physical examination, the overall accuracy of 96.15% is acceptable.

## Conclusions

Our experimental data shows that the TECs of the same persons are highly similar to each other, irrespective of ECG variations induced by the factors such as health status and the ECG electrode placement. The TECs of different persons also show significant differences to each other, compared to self similarity. This feature merits the TEC a good biometric signal for the purpose of authentication. Our algorithm works very well on both datasets of the same health status, and the real-world dataset with no prior knowledge of the health status, by comprehensive evaluations in this study.

Further exploration of our algorithm in a larger and more genetically diversified population [27], [28] will be conducted. The algorithm will also be revised to accommodate the 9 CVD patient samples in Figures 4 and 5 and the 3 healthy individuals in Figure 6 with incorrect predictions in this study.

### Supplementary materials

The algorithm source code and the supplementary materials can be found in the Supplements section of http://www.HealthInformaticsLab.org/supp/. The ECG data may be obtained from the MIT PhysioNet database.

## Supporting Information

Table S1 **Sample IDs used in this study.**
(PDF)
