# Supplementary material for: Normalizing Electrocardiograms of Both Healthy Persons and Cardiovascular Disease Patients for Biometric Authentication
Source: PLoS One. 2013 Aug 20;8(8):e71523. doi: 10.1371/journal.pone.0071523 (PMC3748040; doi:10.1371/journal.pone.0071523)
Supplement: Table S1 — Sample IDs used in this study. (PDF) [file pone.0071523.s001.pdf]

#Sample ID	Database	Annotation
sel100m	QT	Cardiovascular disorder
sel102m	QT	Cardiovascular disorder
sel103m	QT	Cardiovascular disorder
sel104m	QT	Cardiovascular disorder
sel114m	QT	Cardiovascular disorder
sel116m	QT	Cardiovascular disorder
sel117m	QT	Cardiovascular disorder
sel123m	QT	Cardiovascular disorder
sel14046m	QT	Healthy
sel14157m	QT	Healthy
sel14172m	QT	Healthy
sel15814m	QT	Healthy
sel16265m	QT	Healthy
sel16272m	QT	Healthy
sel16273m	QT	Healthy
sel16420m	QT	Healthy
sel16483m	QT	Healthy
sel16539m	QT	Healthy
sel16773m	QT	Healthy
sel16786m	QT	Healthy
sel16795m	QT	Healthy
sel17152m	QT	Cardiovascular disorder
sel17453m	QT	Healthy
sel213m	QT	Cardiovascular disorder
sel221m	QT	Cardiovascular disorder
sel223m	QT	Cardiovascular disorder
sel230m	QT	Cardiovascular disorder
sel231m	QT	Cardiovascular disorder
sel232m	QT	Cardiovascular disorder
sel233m	QT	Cardiovascular disorder
sel30m	QT	Cardiovascular disorder
sel301m	QT	Cardiovascular disorder
sel302m	QT	Cardiovascular disorder
sel306m	QT	Cardiovascular disorder
sel307m	QT	Cardiovascular disorder
sel308m	QT	Cardiovascular disorder
sel31m	QT	Cardiovascular disorder
sel310m	QT	Cardiovascular disorder
sel32m	QT	Cardiovascular disorder
sel33m	QT	Cardiovascular disorder
sel34m	QT	Cardiovascular disorder
sel35m	QT	Cardiovascular disorder
sel36m	QT	Cardiovascular disorder
sel37m	QT	Cardiovascular disorder
sel38m	QT	Cardiovascular disorder
sel39m	QT	Cardiovascular disorder
sel40m	QT	Cardiovascular disorder
sel41m	QT	Cardiovascular disorder
sel42m	QT	Cardiovascular disorder
sel43m	QT	Cardiovascular disorder
sel44m	QT	Cardiovascular disorder
sel45m	QT	Cardiovascular disorder
sel46m	QT	Cardiovascular disorder

#Sample ID	Database	Annotation
sel47m	QT	Cardiovascular disorder
sel48m	QT	Cardiovascular disorder
sel49m	QT	Cardiovascular disorder
sel50m	QT	Cardiovascular disorder
sel51m	QT	Cardiovascular disorder
sel52m	QT	Cardiovascular disorder
sel803m	QT	Cardiovascular disorder
sel808m	QT	Cardiovascular disorder
sel811m	QT	Cardiovascular disorder
sel820m	QT	Cardiovascular disorder
sel821m	QT	Cardiovascular disorder
sel840m	QT	Cardiovascular disorder
sel847m	QT	Cardiovascular disorder
sel853m	QT	Cardiovascular disorder
sel871m	QT	Cardiovascular disorder
sel872m	QT	Cardiovascular disorder
sel873m	QT	Cardiovascular disorder
sel883m	QT	Cardiovascular disorder
sel891m	QT	Cardiovascular disorder
sele0104m	QT	Cardiovascular disorder
sele0106m	QT	Cardiovascular disorder
sele0107m	QT	Cardiovascular disorder
sele0110m	QT	Cardiovascular disorder
sele0111m	QT	Cardiovascular disorder
sele0112m	QT	Cardiovascular disorder
sele0114m	QT	Cardiovascular disorder
sele0116m	QT	Cardiovascular disorder
sele0121m	QT	Cardiovascular disorder
sele0122m	QT	Cardiovascular disorder
sele0124m	QT	Cardiovascular disorder
sele0126m	QT	Cardiovascular disorder
sele0129m	QT	Cardiovascular disorder
sele0133m	QT	Cardiovascular disorder
sele0136m	QT	Cardiovascular disorder
sele0166m	QT	Cardiovascular disorder
sele0170m	QT	Cardiovascular disorder
sele0203m	QT	Cardiovascular disorder
sele0211m	QT	Cardiovascular disorder
sele0303m	QT	Cardiovascular disorder
sele0405m	QT	Cardiovascular disorder
sele0406m	QT	Cardiovascular disorder
sele0409m	QT	Cardiovascular disorder
sele0411m	QT	Cardiovascular disorder
sele0509m	QT	Cardiovascular disorder
sele0603m	QT	Cardiovascular disorder
sele0604m	QT	Cardiovascular disorder
sele0606m	QT	Cardiovascular disorder
sele0607m	QT	Cardiovascular disorder
sele0609m	QT	Cardiovascular disorder
sele0612m	QT	Cardiovascular disorder
sele0704m	QT	Cardiovascular disorder
sele0210m	QT	Cardiovascular disorder
s0306lre	PTB	Healthy

#Sample ID	Database	Annotation
s0303lre	PTB	Healthy
s0302lre	PTB	Healthy
s0291lre	PTB	Healthy
s0311lre	PTB	Healthy
s0312lre	PTB	Healthy
s0273lre	PTB	Healthy
s0287lre	PTB	Healthy
s0301lre	PTB	Healthy
s0299lre	PTB	Healthy
s0322lre	PTB	Healthy
s0275lre	PTB	Healthy
s0328lre	PTB	Healthy
s0274lre	PTB	Healthy
s0304lre	PTB	Healthy
s0305lre	PTB	Healthy
s0300lre	PTB	Healthy
s0374lre	PTB	Healthy
s0308lre	PTB	Healthy
s0363lre	PTB	Healthy
s0336lre	PTB	Healthy
s0402lre	PTB	Healthy
s0436_re	PTB	Healthy
s0452_re	PTB	Healthy
s0457_re	PTB	Healthy
s0460_re	PTB	Healthy
s0461_re	PTB	Healthy
s0462_re	PTB	Healthy
s0465_re	PTB	Healthy
s0466_re	PTB	Healthy
s0467_re	PTB	Healthy
s0468_re	PTB	Healthy
s0469_re	PTB	Healthy
s0471_re	PTB	Healthy
s0472_re	PTB	Healthy
s0473_re	PTB	Healthy
s0474_re	PTB	Healthy
s0478_re	PTB	Healthy
s0479_re	PTB	Healthy
s0481_re	PTB	Healthy
s0486_re	PTB	Healthy
s0487_re	PTB	Healthy
s0491_re	PTB	Healthy
s0496_re	PTB	Healthy
s0499_re	PTB	Healthy
s0500_re	PTB	Healthy
s0502_re	PTB	Healthy
s0504_re	PTB	Healthy
s0526_re	PTB	Healthy
s0527_re	PTB	Healthy
s0531_re	PTB	Healthy
s0543_re	PTB	Healthy

STable1 Sample IDs used in this study.
